# Supplementary material for: Skin temperature and reproductive condition in wild female chimpanzees
Source: PeerJ. 2017 Dec 5;5:e4116. doi: 10.7717/peerj.4116 (PMC5721906; doi:10.7717/peerj.4116)

Skin temperature and reproductive condition in wild female chimpanzees

Guillaume Dezecache, Claudia Wilke, Nathalie Richi, Christof Neumann & Klaus Zuberbühler

19 June 2017

Contents

Data set annotation **2**

Read data and raw data figures **3**

Data preparation **6**

Data analysis **8**

Plot model results **11**

```
# load necessary libraries
library(lme4); library(car); library(effects); library(MuMIn)
sessionInfo()
```

```
## R version 3.3.0 (2016-05-03)
## Platform: x86_64-apple-darwin13.4.0 (64-bit)
## Running under: OS X 10.11.6 (El Capitan)
##
## locale:
## [1] en_US.UTF-8/en_US.UTF-8/en_US.UTF-8/C/en_US.UTF-8/en_US.UTF-8
##
## attached base packages:
## [1] stats     graphics  grDevices utils     datasets  methods   base
##
## other attached packages:
## [1] MuMIn_1.15.6   effects_3.1-2  car_2.1-4      lme4_1.1-12    Matrix_1.2-7.1
##
## loaded via a namespace (and not attached):
##  [1] Rcpp_0.12.10       knitr_1.15.20      magrittr_1.5       splines_3.3.0
##  [5] MASS_7.3-45        colorspace_1.3-1   lattice_0.20-34    minqa_1.2.4
##  [9] stringr_1.2.0      tools_3.3.0        nnet_7.3-12        pbkrtest_0.4-6
## [13] parallel_3.3.0     grid_3.3.0         nlme_3.1-128       mgcv_1.8-16
## [17] quantreg_5.29      MatrixModels_0.4-1 htmltools_0.3.6    yaml_2.1.14
## [21] rprojroot_1.2      digest_0.6.12      nloptr_1.0.4       evaluate_0.10
## [25] rmarkdown_1.5      stringi_1.1.5      backports_1.0.5    stats4_3.3.0
## [29] SparseM_1.74
```

Data set annotation

The data for this study are in `chimpskintemperature.csv`. The data set contains the following columns:

- **Date** The calendar date the image was recorded.
- **fem** Subject ID
- **img** Image ID.
- **temper** Subject skin temperature (°Celsius).
- **humid** Humidity (percent).
- **dist** Distance between camera and subject (meters).
- **ambitemp** Ambient temperature (°Celsius).
- **bodypart** Bodypart of the subject.
- **preg** Was the female pregnant (1) or cycling (0).
- **swell** The swelling stage of the female (0 through 4).

Read data and raw data figures

```
xdata <- read.table("chimpskintemperature.csv", header=T, sep=",")

mypars <- par(no.readonly = T) # save graphical parameters

# settings for graphical adjustments (separation, jitter etc)
offs <- 0.15 # separation of groups
offs2 <- 0.05 # for point jitter
offs3 <- 0.1 # for median width

# create layout
lmat <- matrix(c(10, 1, 2, 3, 12,
               10, 4, 5, 6, 12,
               10, 7, 8 ,9, 12,
               13, 11, 11, 11, 14), ncol = 5, byrow = T)
layout(mat = lmat, widths = c(1, 4, 4, 4, 2), heights = c(4, 4, 4, 1))

# loop through each bodypart
for(f in levels(xdata$bodypart)) {
  # set up empty plot
  par(mar = c(0.7, 1, 1.7, 0.1))
  plot(0, 0, "n", xlim = c(-0.5, 4.5), ylim = c(18, 38), ann = F, axes = F,
       yaxs = "i", xaxs = "i")

  # extract relevant data points
  temp <- xdata[xdata$bodypart == f, ]
  preg <- temp[temp$preg == 1, ]
  cycl <- temp[temp$preg == 0, ]

  # plot points for cycling and swelling
  x <- preg$swell + offs +
    runif(n = length(preg$swell), min = offs2*(-1), max = offs2) # add some jitter
  points(x, preg$temper, pch = 16, col = rgb(0.2, 0.2, 0.2, 0.3), cex = 0.5)
  x <- cycl$swell - offs +
    runif(n = length(cycl$swell), min = offs2*(-1), max = offs2) # add some jitter
  points(x, cycl$temper, pch = 16, col = rgb(1, 0, 0, 0.3), cex = 0.5)

  # plot medians and quartiles
  x <- tapply(preg$temper, preg$swell, median)
  xx <- as.numeric(names(x))
  segments(x0 = xx + offs - offs3, y0 = x, x1 = xx + offs + offs3, y1 = x,
           col = rgb(0, 0, 0), lwd=2)
  x1 <- tapply(preg$temper, preg$swell, quantile, probs = 0.25)
  x2 <- tapply(preg$temper, preg$swell, quantile, probs = 0.75)
  segments(x0 = xx + offs, y0 = x1, x1 = xx + offs, y1 = x2,
           col = rgb(0, 0, 0), lwd=1)

  x <- tapply(cycl$temper, cycl$swell, median)
```

```r
  xx <- as.numeric(names(x))
  segments(x0 = xx - offs - offs3, y0 = x, x1 = xx - offs + offs3, y1 = x,
           col = rgb(1, 0, 0), lwd = 2)
  x1 <- tapply(cycl$temper, cycl$swell, quantile, probs = 0.25)
  x2 <- tapply(cycl$temper, cycl$swell, quantile, probs = 0.75)
  segments(x0 = xx - offs, y0 = x1, x1 = xx - offs, y1 = x2,
           col = rgb(1, 0, 0), lwd = 1)

  # add axes
  if(f %in% c("Ear", "Foot", "Neck")) {
    axis(2, tcl = -0.18, xpd = T, las = 1, labels = NA)
    v <- c(20, 25, 30, 35)
    text(labels =  v, x = -0.68, y = v, xpd = T, cex = 0.8)
  }

  if(f %in% c("Neck", "Nose", "Swelling")) {
    mtext(text = 0:4, side = 1, line = 0.3, xpd = T, at = 0:4, cex = 0.5)
  }

  # add body part labels
  box()
  rect(-0.5, 38.5, 4.5, 40.5, xpd=T, border = NA, col = "grey")
  text(2, 39.5, labels = f, xpd = T)

}

# add overall axes annotations
par(mar = c(0, 0, 0, 0))
plot(0, 0, "n", axes = F, ann = F)
bq <- bquote(plain("surface temperature (")~degree~plain("Celsius)"))
text(-0.1, 0, labels = bq , srt = 90, cex = 1.5)

plot(0, 0, "n", axes = F, ann = F)
text(0, 0, labels = "swelling stage", cex = 1.5)

# legend
plot(0,0,"n", axes=F, ann=F)
legend("center", col = c("red", "black"), legend = c("cycling", "pregnant"),
       pch = 16, pt.cex = 1.2, bty = "n")

par(mypars) # reset graphical parameters
```

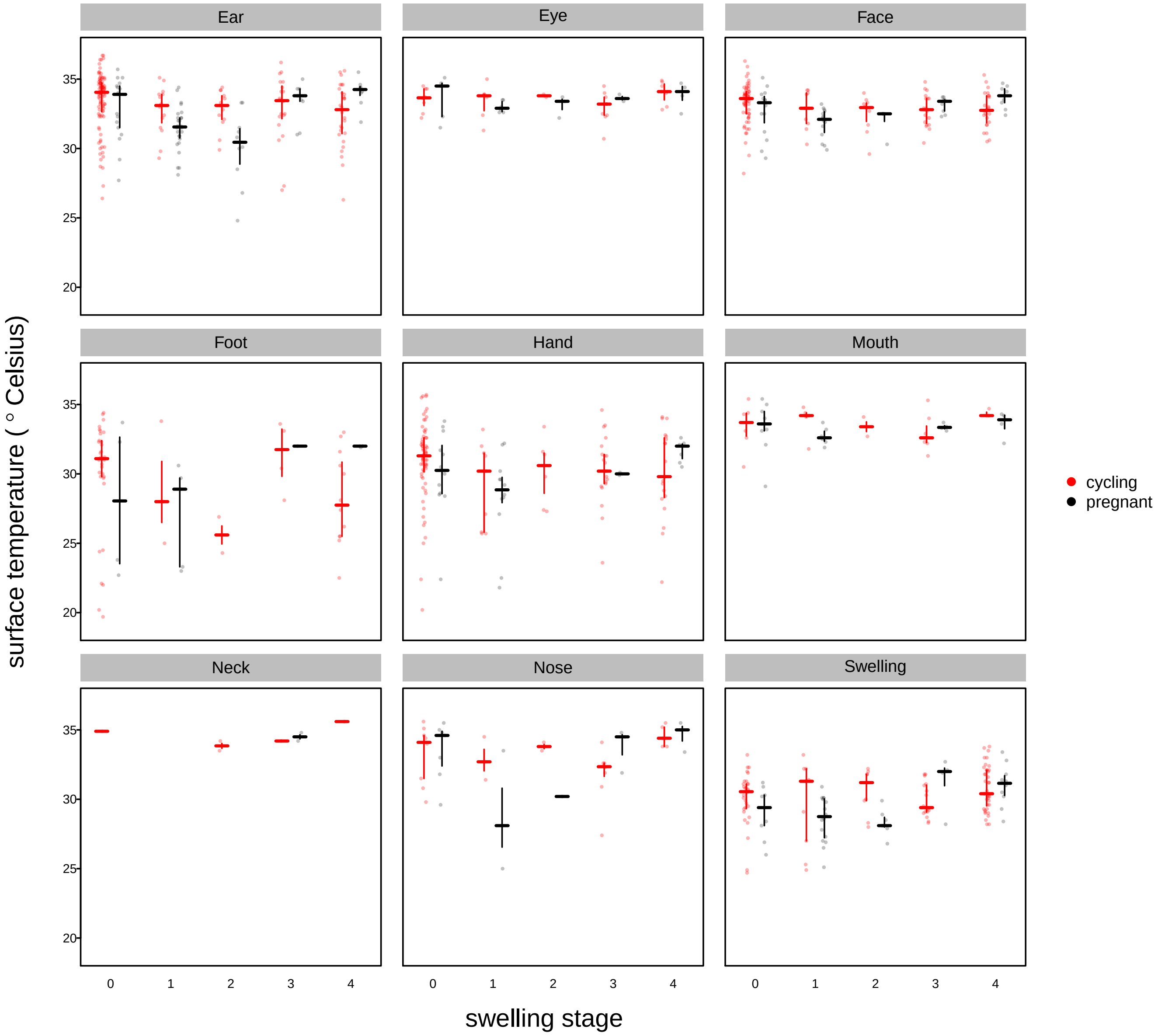
Ear
Eye
Face
Foot
Hand
Mouth
Neck
Nose
Swelling
surface temperature (° Celsius)
swelling stage
20
25
30
35
0
1
2
3
4
cycling
pregnant

Data preparation

```
# check distributions, transform if necessary and standardize
tempermin <- min(xdata$temper) # keep minimum temperature (for back transforming in the figures)
par(mfcol = c(1, 2))
hist(xdata$temper)
hist((xdata$temper - tempermin)^2)
```

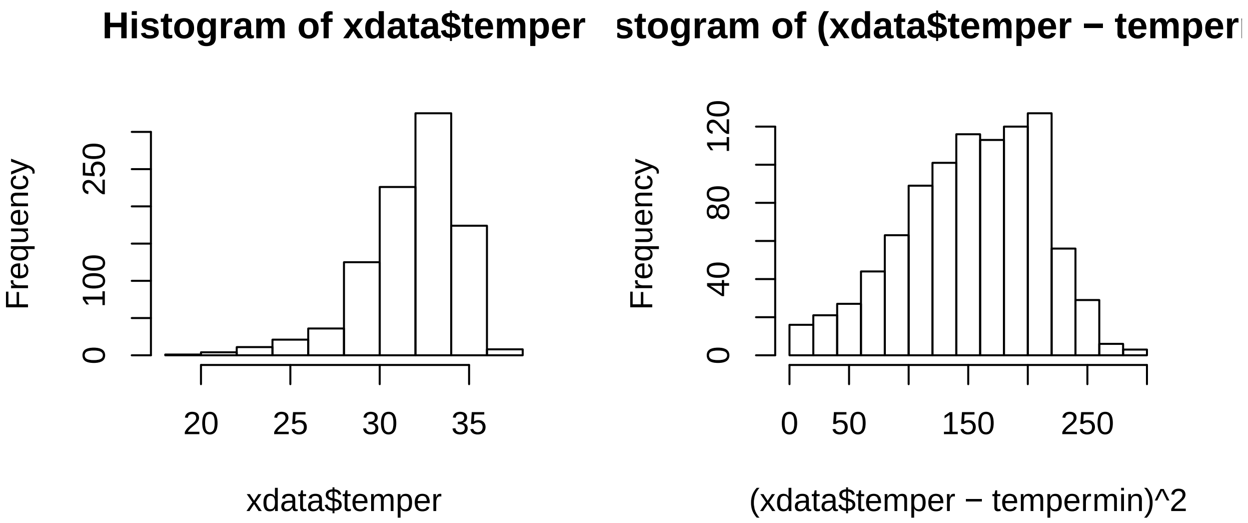


```
xdata$temper <- (xdata$temper - tempermin)^2

hist(xdata$ambitemp)
hist((xdata$ambitemp - min(xdata$ambitemp))^2)
```

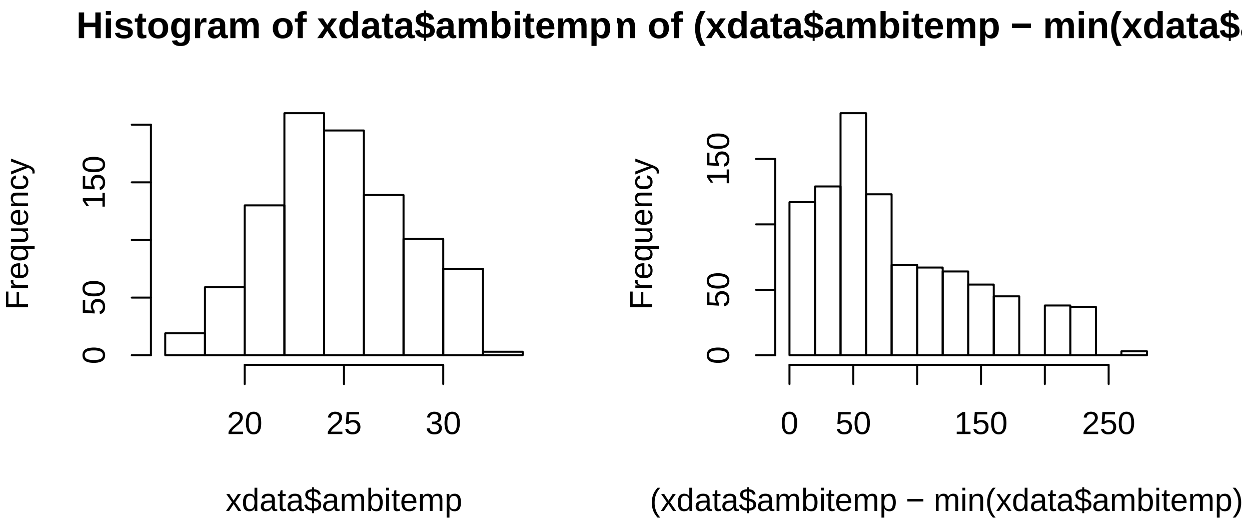


```
xdata$ambitemp <- scale(xdata$ambitemp)

hist(xdata$dist)
hist(sqrt(xdata$dist))
```

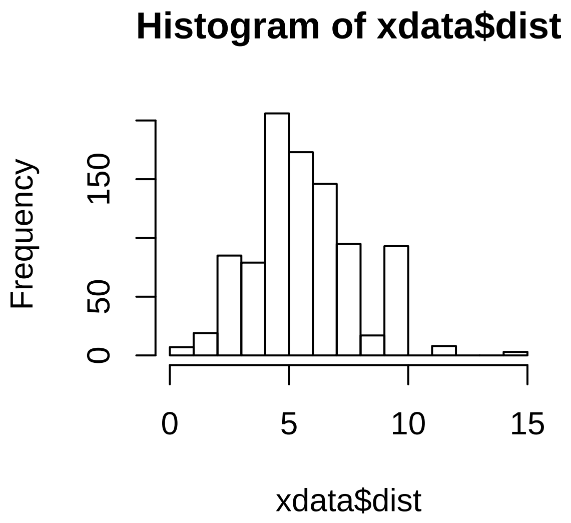

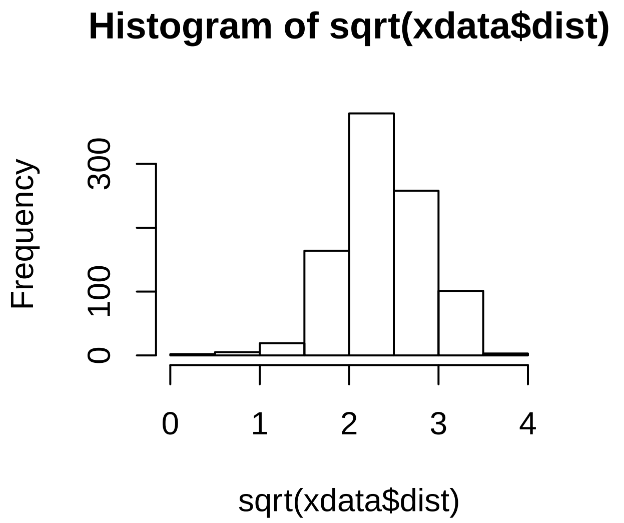


```
xdata$dist <- scale(sqrt(xdata$dist))

hist(xdata$humid)
hist((xdata$humid - min(xdata$humid))^2)
```

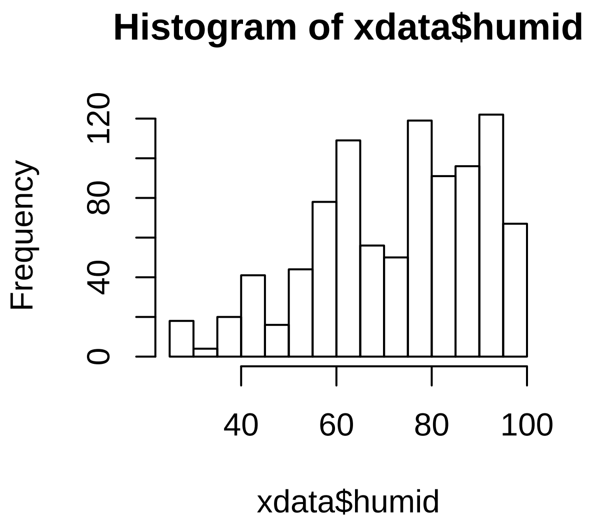

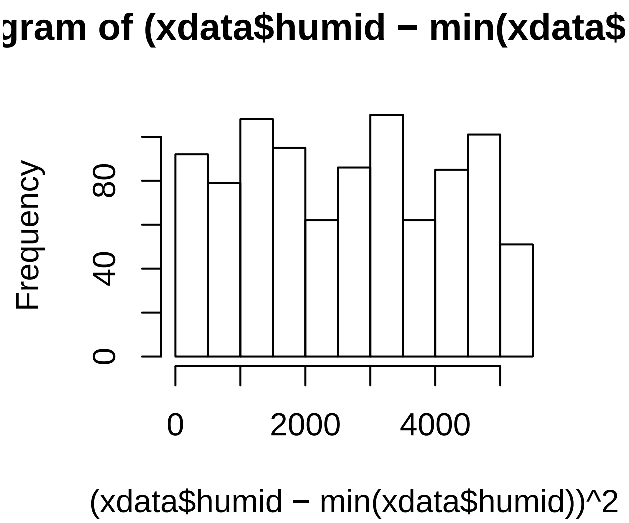


```
xdata$humid <- scale((xdata$humid - min(xdata$humid))^2)

# recode swelling as factor
xdata$swell <- as.factor(paste0("s", as.character(xdata$swell)))
```

Data analysis

```
# fit full model
res <- lmer(temper ~ swell*preg +ambitemp +dist +humid
            +(swell +preg|bodypart) +(1|fem/img), data=xdata, REML=F)

# diagnostics and assumption checks
par(mfrow = c(3, 2))
VarCorr(res)
```

```
##  Groups   Name        Std.Dev. Corr
##  img:fem  (Intercept) 28.0561
##  fem      (Intercept) 13.2111
##  bodypart (Intercept) 31.5690
##           swells1      4.5311   0.338
##           swells2     11.9758   0.803  0.275
##           swells3      2.3569  -0.172 -0.571 -0.630
##           swells4     12.5407   0.665 -0.272  0.847 -0.260
##           preg         2.1850   0.200  0.970  0.044 -0.392 -0.480
##  Residual             19.6363
```

```
vif(lm(temper ~ swell +preg +ambitemp +dist +humid, data=xdata))
```

```
##              GVIF Df GVIF^(1/(2*Df))
## swell    1.257426  4        1.029047
## preg     1.115712  1        1.056273
## ambitemp 2.748825  1        1.657958
## dist     1.058186  1        1.028682
## humid    2.674543  1        1.635403
```

```
hist(resid(res))
plot(fitted(res), resid(res))
qqnorm(resid(res)); qqline(resid(res))
plot(resid(res) ~ xdata$swell)
plot(resid(res) ~ as.factor(xdata$preg))

# fit null model
null <- lmer(temper ~  ambitemp +dist +humid
             +(swell+ preg|bodypart) +(1|fem/img), data=xdata, REML=F)

# compare null and full model (likelihood ratio test)
anova(null, res, test = "Chisq")
```

```
## Data: xdata
## Models:
## null: temper ~ ambitemp + dist + humid + (swell + preg | bodypart) +
## null:     (1 | fem/img)
## res: temper ~ swell * preg + ambitemp + dist + humid + (swell + preg |
## res:     bodypart) + (1 | fem/img)
##      Df    AIC    BIC  logLik deviance  Chisq Chi Df Pr(>Chisq)
## null 28 9259.1 9394.5 -4601.6   9203.1
```

```
## res  37 9257.6 9436.6 -4591.8   9183.6 19.483      9    0.02139 *
## ---
## Signif. codes:  0 '***' 0.001 '**' 0.01 '*' 0.05 '.' 0.1 ' ' 1
```

```
# test interaction
red <- update(res, .~. -preg:swell)
anova(red, res, test = "Chisq")
```

```
## Data: xdata
## Models:
## red: temper ~ swell + preg + ambitemp + dist + humid + (swell + preg |
## red:     bodypart) + (1 | fem/img)
## res: temper ~ swell * preg + ambitemp + dist + humid + (swell + preg |
## res:     bodypart) + (1 | fem/img)
##     Df    AIC    BIC  logLik deviance Chisq Chi Df Pr(>Chisq)
## red 33 9259.3 9418.9 -4596.7   9193.3
## res 37 9257.6 9436.6 -4591.8   9183.6 9.717      4    0.04547 *
## ---
## Signif. codes:  0 '***' 0.001 '**' 0.01 '*' 0.05 '.' 0.1 ' ' 1
```

```
# R2
r.squaredGLMM(res)
```

```
##      R2m      R2c
## 0.328893 0.899165
```

```
# show full model results
coefficients(summary(res))
```

```
##                 Estimate Std. Error    t value
## (Intercept)  168.149071  11.435515 14.7041099
## swells1       -7.438109   6.908385 -1.0766784
## swells2      -13.939526   7.773089 -1.7933060
## swells3       -3.717200   5.384479 -0.6903547
## swells4      -12.258746   6.522388 -1.8794874
## preg         -11.315313   6.330055 -1.7875536
## ambitemp      29.651874   2.165204 13.6947240
## dist          -4.662080   1.382377 -3.3725110
## humid         -4.892678   2.209884 -2.2139974
## swells1:preg   2.355971   8.984847  0.2622160
## swells2:preg -19.811164  10.822659 -1.8305264
## swells3:preg  17.624968  10.315699  1.7085578
## swells4:preg   9.978287   9.302887  1.0726012
```

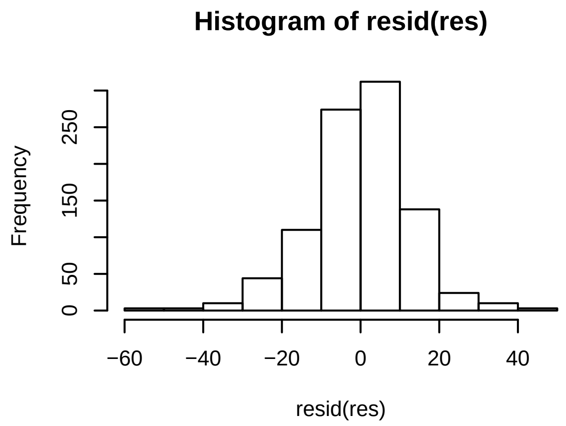
Histogram of resid(res)
Frequency
0
50
150
250
−60
−40
−20
0
20
40
resid(res)

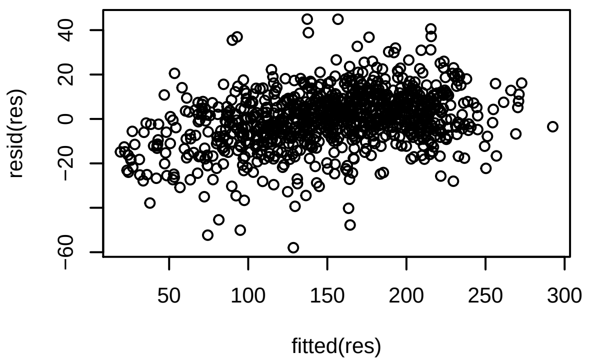
resid(res)
40
20
0
−20
−60
50
100
150
200
250
300
fitted(res)

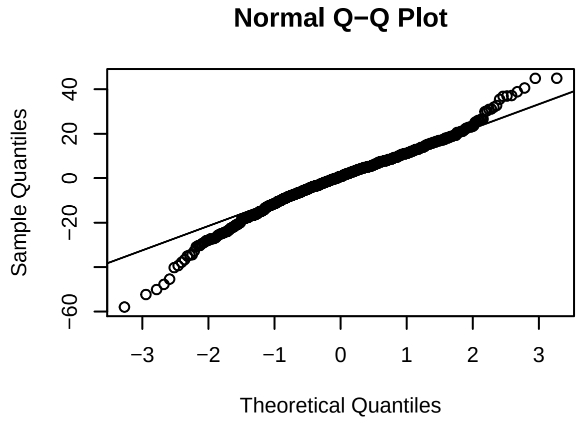
Normal Q−Q Plot
Sample Quantiles
40
20
0
−20
−60
−3
−2
−1
0
1
2
3
Theoretical Quantiles

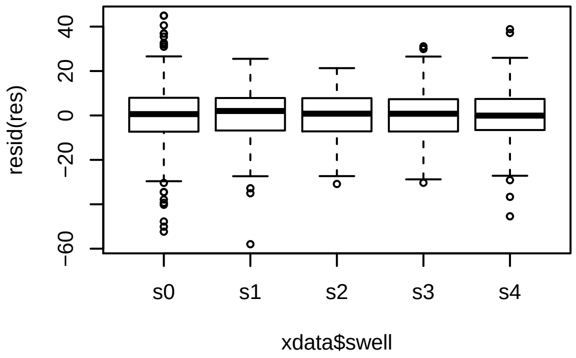
resid(res)
40
20
0
−20
−60
s0
s1
s2
s3
s4
xdata$swell

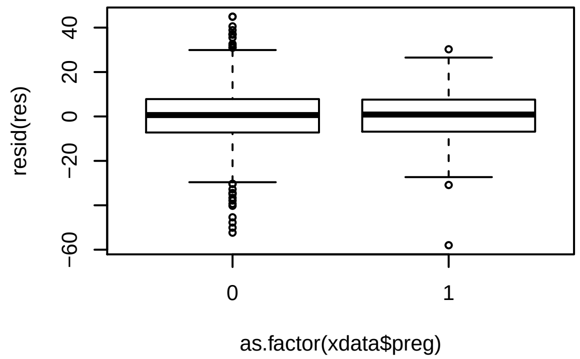
resid(res)
40
20
0
−20
−60
0
1
as.factor(xdata$preg)

Plot model results

```
# create plotting data (via effects package)
pdatares <- data.frame(effect("swell:preg", res, xlevels = list(preg = c(0, 1))))

# draw empty plot without axes
bq <- bquote(plain("surface temperature (")~degree~plain("Celsius)"))
plot(0, 0, xlim = c(0.5, 5.5), ylim = c(45, 220), xlab = "swelling stage",
     ylab = bq, type = "n", axes = F)

# make x-coordinates
xvals <- c(1:5, 1:5) + rep(c(-0.18, 0.18), each = 5)

# add point estimates and CIs
points(xvals, pdatares$fit, pch = rep(c(15, 16), each = 5),
       col = rep(c("red", "black"), each = 5))
arrows(xvals, pdatares$lower, xvals, pdatares$upper, code = 3, angle = 90, length = 0,
       col = rep(c("red", "black"), each = 5))
box()

# draw axes
axis(1, at = c(1:5), labels = 0:4)
labs <- seq(25, 35, by = 1)
ats <- (labs - tempermin)^2  # backtransform to original scale
axis(2, las = 1, at = ats, labels = labs)

# legend
legend("topleft", legend = c("cycling", "pregnant"), col = c( "red", "black"),
       pch = c(15, 16), horiz = T, bty = "n", pt.cex = 1.3, cex = 0.9)
```

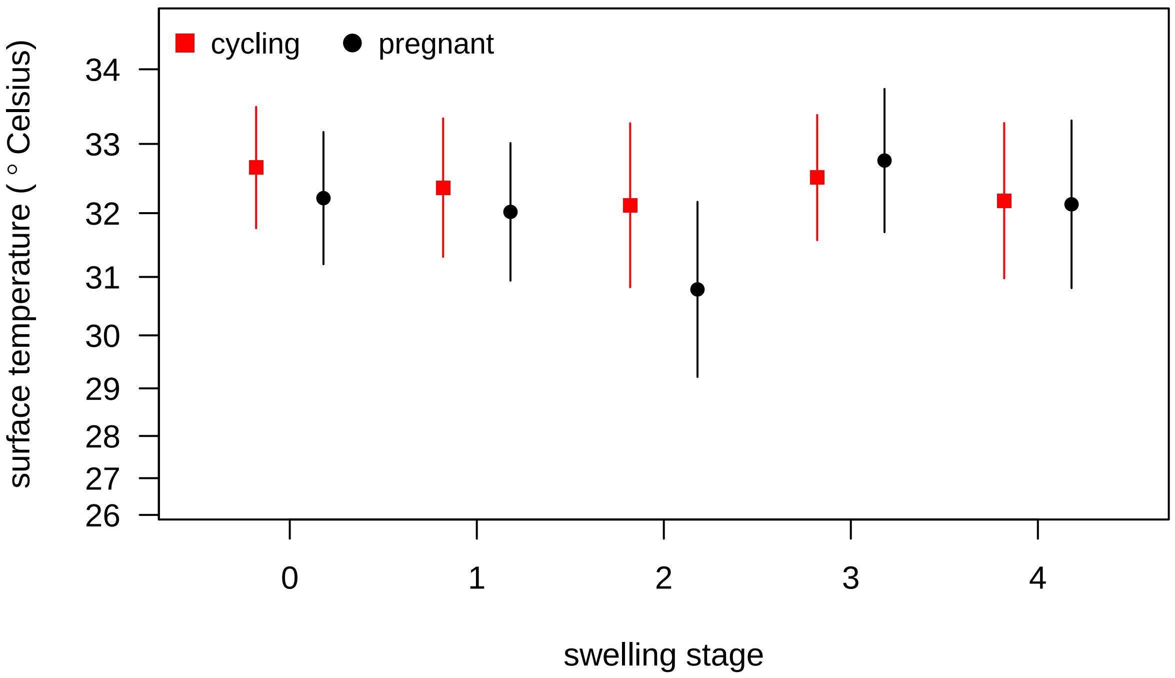

```r
# create new data frame with predicted values
newd <- data.frame(expand.grid(bodypart = levels(xdata$bodypart),
                               swell = levels(xdata$swell), preg = c(0, 1)),
                   humid = 0, dist = 0, ambitemp = 0, fem = NA, img = NA)
newd$pred <- predict(res, newd, re.form = ~(swell +preg|bodypart))

# a function to plot per body part
plotfunc <- function(bpart, MAIN, YLIM, legendpos = "bottomleft", xdata = newd) {
  # select bodypart
  pdata <- xdata[xdata$bodypart == bpart, ]
  # cycling first, leftish (to the right: pregnant)
  pdata$xcord <- rep(1:5, 2) + rep(c(-0.15, 0.15), each = 5)
  # cycling = red, pregnant = black
  pdata$co <- rep(c("red", "black"), each = 5)
  # cycling = square, pregnant = circle
  pdata$ps <- rep(c(15, 16), each = 5)
  # set up plot and add axes
  bq <- bquote(plain("surface temperature (")~degree~plain("Celsius)"))
  plot(0, 0, xlim = c(0.5, 5.5), ylim = YLIM, "n", axes = F, xlab = "swelling stage",
       ylab = bq, main = MAIN)
  axis(1, at = 1:5, labels = 0:4,lwd = NA); box()
  # y-axis: backtransform
  labs <- seq(25, 35, by = 1)
  ats <- (labs - tempermin) ^ 2
  axis(2, at = ats, labels = labs, las = 1)

  # separate swelling stages by lines
  abline(v = seq(0.5, 5.5, by = 1), lty = 3, col = "grey80")

  # plot point estimates
  points(pdata$xcord, pdata$pred, pch=pdata$ps, col = pdata$co, cex = 1.5)

  # add legend
  legend(legendpos, legend = c("cycling", "pregnant"), pch = c(15, 16), bty = "n",
         col = c("red", "black"), cex = 0.8)
}


YLIMS <- c(45, 220) # on the transformed scale

par(mfrow = c(3, 3))
plotfunc("Ear", "ear", YLIMS)
plotfunc("Eye", "eye", YLIMS)
plotfunc("Face", "face", YLIMS)
plotfunc("Foot", "foot", YLIMS)
plotfunc("Hand", "hand", YLIMS)
plotfunc("Mouth", "mouth", YLIMS)
plotfunc("Neck", "neck", YLIMS)
plotfunc("Nose", "nose", YLIMS)
```

```
plotfunc("Swelling", "swelling", YLIMS)
```

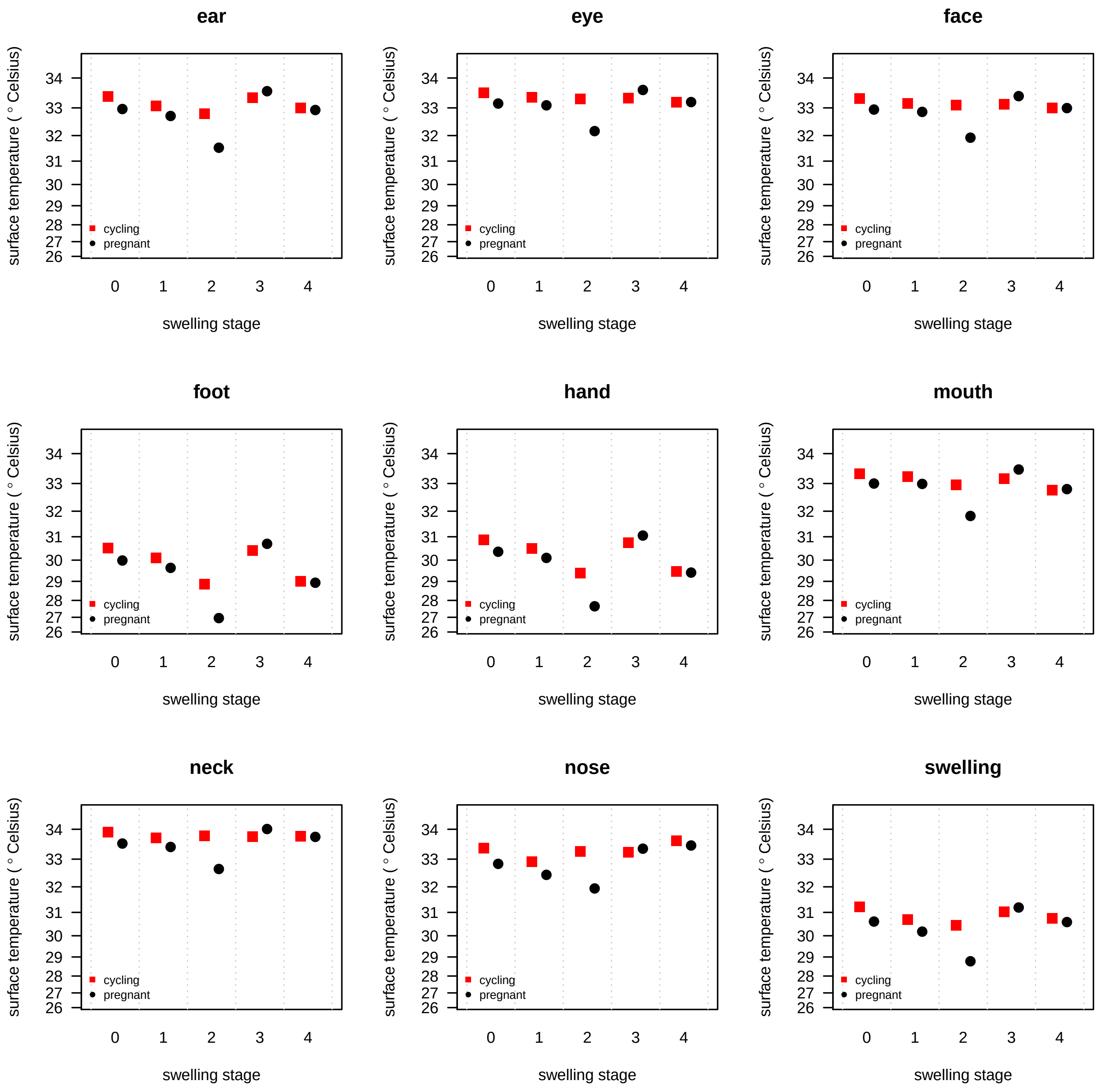

Supplement: Supplemental Information 2 — Supporting information for the interpretation and use of the raw data and analysis script. [file peerj-05-4116-s003.pdf]
